# Sequential chemo-durvalumab, reduced-dose RT, and consolidation durvalumab for unresectable stage III NSCLC unfit for PACIFIC regimen (DEDALUS trial)

**DOI:** 10.1093/jncics/pkag050

**Published:** 2026-05-06

**Authors:** Francesco Agustoni, Jessica Saddi, Diego Luigi Cortinovis, Stefano Arcangeli, Luca Sala, Catherine Klersy, Valeria Musella, Giulia Galli, Sabrina Maria Chiara Borgetto, Giulia Maria Stella, Daniela Cicognini, Alessandra Ferrari, Paolo Pedrazzoli, Francesco Grossi, Andrea Riccardo Filippi

**Affiliations:** Department of Internal Medicine and Medical Therapeutics, University of Pavia, Pavia, Italy; Department of Oncology—Comprehensive Cancer Center, Fondazione IRCCS Policlinico San Matteo, Pavia, Italy; Radiation Oncology, Fondazione IRCCS Policlinico San Matteo, Pavia, Italy; Department of Medicine, University Milano-Bicocca, Milan, Italy; Medical Oncology Unit, Fondazione IRCCS San Gerardo dei Tintori, Monza, Italy; Department of Medicine and Surgery, University Milano-Bicocca, Milan, Italy; Radiation Oncology Unit, Fondazione IRCCS San Gerardo dei Tintori, Monza, Italy; Medical Oncology Unit, Fondazione IRCCS San Gerardo dei Tintori, Monza, Italy; Biostatistics and Clinical Trial Center, Fondazione IRCCS Policlinico San Matteo, Pavia, Italy; Biostatistics and Clinical Trial Center, Fondazione IRCCS Policlinico San Matteo, Pavia, Italy; Department of Oncology—Comprehensive Cancer Center, Fondazione IRCCS Policlinico San Matteo, Pavia, Italy; Department of Oncology—Comprehensive Cancer Center, Fondazione IRCCS Policlinico San Matteo, Pavia, Italy; Department of Internal Medicine and Medical Therapeutics, University of Pavia, Pavia, Italy; Department of Medical Sciences and Infective Disease, Unit of Respiratory Disease, Fondazione IRCCS Policlinico San Matteo, Pavia, Italy; Department of Oncology—Comprehensive Cancer Center, Fondazione IRCCS Policlinico San Matteo, Pavia, Italy; Department of Oncology—Comprehensive Cancer Center, Fondazione IRCCS Policlinico San Matteo, Pavia, Italy; Department of Internal Medicine and Medical Therapeutics, University of Pavia, Pavia, Italy; Department of Oncology—Comprehensive Cancer Center, Fondazione IRCCS Policlinico San Matteo, Pavia, Italy; IRCCS Ospedale Policlinico San Martino, Genova, Italy; Medical Oncology Division, ASST Sette Laghi, Varese, Italy; Department of Oncology, University of Milan, Milan, Italy; Radiation Oncology, Fondazione IRCCS Istituto Nazionale dei Tumori, Milan, Italy

## Abstract

**Background:**

The PACIFIC study established the standard of care for unresectable, stage III non-small cell lung cancer (NSCLC). The DEDALUS trial is a phase 2, open-label, multicenter study enrolling patients who are eligible for sequential chemo-radiation therapy (CRT) plus immunotherapy.

**Methods:**

Patients had unresectable stage IIIA-C NSCLC, regardless of PD-L1 status. After 3 cycles of chemo-durvalumab, responders received hypo-fractionated thoracic radiotherapy (45 Gy over 3 weeks) with durvalumab, then continued durvalumab for up to 12 months or until disease progression. Primary endpoint was safety, assessed by the incidence of grade 3 and 4 possibly related adverse events (PRAEs) within 6 months. Secondary endpoints included progression-free survival (PFS), overall survival (OS), and quality of life (NCT05128630).

**Results:**

Between February 2022 and August 2024, 28 patients were screened, and 25 enrolled across 3 Italian centers. Enrollment was halted early due to low recruitment. We recorded 9 grade 3-4 PRAEs, which accounted for 6.4% of all AEs; 7 patients (28%) experienced at least 1 grade 3-4 PRAE. Only 1 was immune-related, while the remaining PRAEs were related to chemotherapy, none to RT. Median PFS was 13.2 months (95% CI = 4.9 to 18.6), median OS was 17.5 months (95% CI = 10.7 to 18.6). Among the 16 patients who started maintenance without progression median PFS was 18.6 months (95% CI = 12.8 to not reached), median OS was not reached.

**Conclusions:**

The early closure of the study and the reduced sample size make it difficult to draw significant conclusions. However, feasibility and safety seem to be acceptable, and early PFS and OS data are promising, especially for patients who completed the full treatment sequence.

## Introduction

Non-small cell lung cancer (NSCLC) accounts for approximately 85% of all lung cancers.^1^ Around 30% of NSCLC cases are diagnosed at stage III, which is considered locally advanced disease.^2^ Stage III covers a broad range of presentations, marked by tumor growth into nearby structures and/or regional lymph node involvement without distant metastases. Patients often present with cardiopulmonary comorbidities, are over 70 years old, and show signs of frailty or vulnerability.^3^

The standard of care is concurrent platinum-based chemoradiation (cCRT) at 60-66 Gy delivered in 30-33 fractions over 6 weeks, followed by durvalumab maintenance for responders.^4^^,^^5^ This approach yields a median progression-free survival (PFS) of 16.9 months, a median overall survival (OS) of 47.5 months (95% CI = 38.1 to 52.9 months), and a 5-year OS rate of 42.9%.^6-8^ The effectiveness of the PACIFIC regimen is the result of the independent effects of chemoradiation, anti-PD-L1 agents, and their interaction, which is believed to be a key mechanism.^9^^,^^10^ However, the exact process remains unclear, as this therapeutic sequence is highly effective in only about half of the patients, depending on PD-L1 expression levels.

Studies in real-world settings have shown that not all patients are suitable for cCRT. The PACIFIC-R, a global retrospective study of around 1300 patients, also included some patients who received sequential CRT (sCRT).^11^^,^^12^ This study found that patients who received cCRT had slightly better PFS and OS. The PACIFIC-6 phase 2 trial was designed to test the effectiveness of sCRT plus durvalumab and confirmed that this treatment is safe and effective.^13^^,^^14^ Also the GEMSTONE-301 phase 3 trial assessed the efficacy and safety of sugemalimab, an anti-PD-L1 antibody, in patients with stage III NSCLC whose disease had not progressed after concurrent or sequential chemo-radiotherapy; in particular 33% of enrolled patients received sCRT and 67% cCRT, the PFS HR was 0.59 (95% CI = 0.39 to 0.91) and 0.66 (95% CI = 0.44 to 0.99), respectively.^15^

CT, in combination with immune checkpoint inhibitors (ICIs), currently represents the mainstay of treatment for patients with locally advanced disease not suitable for local or radical therapy, as well as for those with advanced disease, and may elicit greater activity and efficacy than chemotherapy alone. For these reasons, we developed the DEDALUS phase 2 trial to evaluate a new approach that combines induction chemo-immunotherapy, followed by reduced-dose, hypo-fractionated thoracic RT administered concurrently and subsequently with immunotherapy maintenance in patients with stage 3 unresectable NSCLC who are candidates for sCRT (like in PACIFIC-6). DEDALUS differs from PACIFIC-6 by including induction chemo-immunotherapy and reducing the radiation dose from 60 to 45 Gy.

We hypothesize that this new regimen may leverage the benefits of induction chemo-immunotherapy, potentially increasing response rates, allowing for a lower RT dose without sacrificing tumor control, and thereby reducing radiation-induced immunosuppression and late radiation-related morbidity.

## Methods

### Trial design and patient disposition

DEDALUS is a phase 2, open-label, single-arm, multicenter academic trial (NCT05128630), sponsored by Fondazione IRCCS Policlinico San Matteo, Pavia, Italy.

Patients needed to have histologically or cytologically confirmed NSCLC and unresectable stage III disease as defined by the International Association for the Study of Lung Cancer (IASLC) Staging Manual, Version 8 (IASLC 2016).^16^

After multidisciplinary discussion and clinical evaluation by the medical oncologist and radio-oncologist, patients were considered ineligible for concurrent chemo-radiotherapy.

All patients had past or present medical conditions at baseline. Overall, 72% had a history of cardiovascular disorders (primarily hypertension, reported in 36% of all enrolled patients). A total of 16% had a history of respiratory disorders, 20% of gastrointestinal disorders, 4% of diabetes mellitus, and 20% of neurological disorders (Table 1).

**Table 1.** Baseline patient and disease characteristics in the intention-to-treat (ITT) and per-protocol (PP) populations.

Variables	ITT population, No. (%) (*n* = 25)	PP population, No. (%) (*n* = 16)
Age	70.4 (SD 8.2) Range: 51-84	69.4 (SD 7.7) Range: 57-84
Sex		
Male	18 (72.0)	11 (68.8)
Female	7 (28.0)	5 (31.1)
ECOG performance status		
0	8 (32.0)	7 (43.8)
1	15 (60.0)	8 (50.0)
≥2	2(8.0)	1 (6.2)
Smoking status		
Never smoker	0 (0.0)	0 (0.0)
Current smoker	9 (36.0)	6 (37.5)
Former smoker	16 (64.0)	10 (62.5)
Tumor histological type		
Non-squamous	12 (48.0)	8 (50.0)
Squamous	12 (48.0)	7 (43.8)
Other	1 (4.0)	1 (6.2)
Comorbidities		
Cardiopathy	9 (36.0)	3 (18.8)
Hypertension	9 (36.0)	6 (37.5)
Diabetes	1 (4.0)	1 (6.2)
Liver failure	0 (0.0)	0 (0.0)
Kidney failure	0 (0.0)	0 (0.0)
Lung failure	4 (16.0)	3 (18.8)
Gastrointestinal disease	5 (20.0)	5 (31.2)
Neurological disease	3 (12.0)	2 (12.5)
PD-L1 TPS status		
<1%	8 (32.0)	5 (31.3)
≥1%	15 (60.0)	10 (62.5)
Unknown	2 (8.0)	1 (6.2)
Disease stage (T)		
T1	5 (20.0)	4 (25.0)
T2	4 (16.0)	3 (18.8)
T3	6 (24.0)	3 (18.8)
T4	7 (28.0)	5 (31.2)
Unknown	3 (12.0)	1 (6.2)
Disease stage (N)		
N1	2 (8.0)	2 (12.5)
N2	13 (52.0)	8 (50.0)
N3	7 (28.0)	5 (31.3)
Unknown	3 (12.0)	1 (6.2)
Disease stage (TNM)		
IIIA	8 (32.0)	6 (37.5)
IIIB	10 (40.0)	7 (43.8)
IIIC	4 (16.0)	2 (12.5)
Unknown	3 (12.0)	1 (6.2)

**Figure 1.**
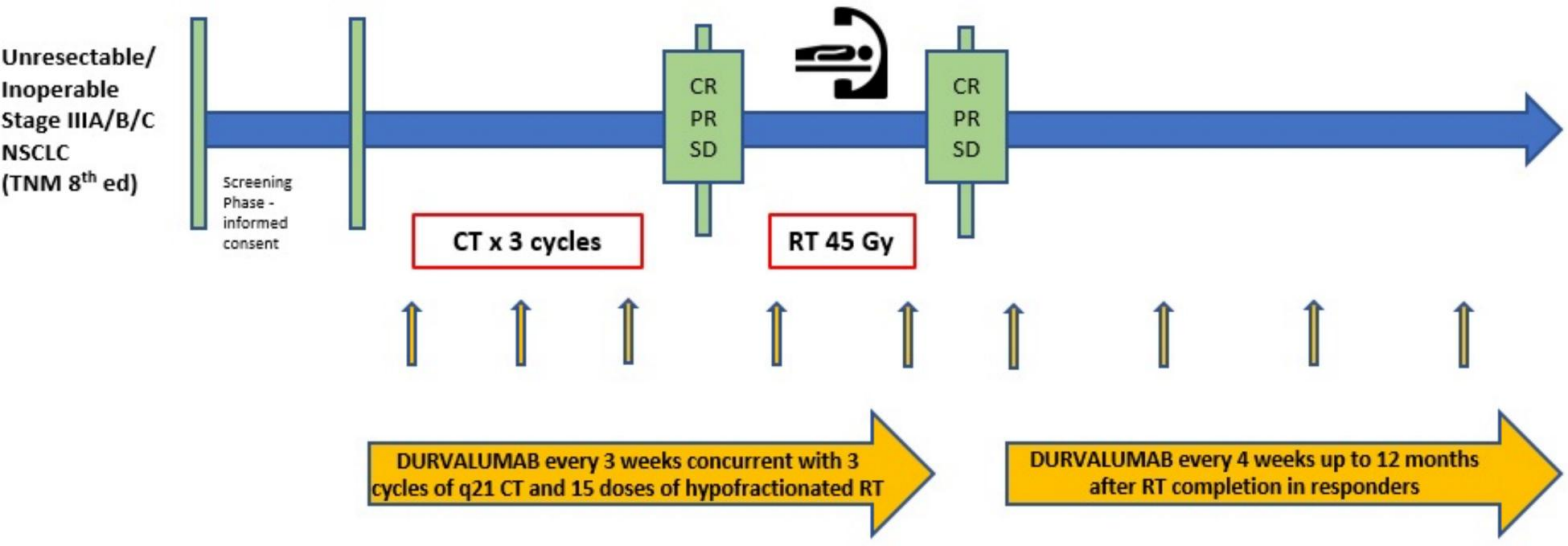
Trial design. Abbreviations: CR = complete response; CT = chemotherapy; NSCLC = non-small cell lung cancer; PR = partial response; RT = radiotherapy; SD = stable disease.

Patients deemed eligible for sCRT by the multidisciplinary team at the treating center were enrolled. After an induction phase consisting of 3 cycles of chemotherapy (cisplatin/carboplatin + etoposide) plus durvalumab, responding patients (stable disease—SD, complete response—CR, partial response—PR), as assessed by the investigators, received reduced-dose hypo-fractionated thoracic radiotherapy (45 Gy in 3 Gy fractions over 3 weeks) plus durvalumab, followed by durvalumab maintenance up to 12 months. The trial design and treatment sequence are illustrated in Figure 1.

Each patient was asked to provide a written informed consent before entering the study, and the study was conducted following approval by the Ethical Committee and Study Protocols Review Board of the Fondazione IRCCS Policlinico San Matteo Hospital. All procedures used in this study comply with the 1975 Declaration of Helsinki.^17^

### Chemo-immunotherapy

CT consisted of administering etoposide at 80-100 mg/m^2^ through IV infusion on days 1-3 of each 21-day cycle and either carboplatin at an area under the curve of 5-6 mg/mL/min or cisplatin at 75-80 mg/m^2^ on day 1 of each cycle of 3 full 21-day cycles. Durvalumab was administered intravenously at a fixed dose of 1500 mg every 3 weeks during concurrent chemotherapy, at the same interval during radiotherapy (RT), and then every 4 weeks during maintenance, for up to 12 months or until unacceptable toxicity, patient withdrawal, or other discontinuation criteria were met.

### Radiotherapy

Tumor and lymph nodes were targeted using standard protocols. All targets were contoured adjusting pre-induction lesions; all patients were planned using VoluMetric Arc Therapy (VMAT) technique. We predefined dose constraints for organs at risk and adjusted them for the slightly hypo-fractionated 45 Gy regimen, given in 3 Gy daily fractions.

### Endpoints and assessment

The primary endpoint was to assess safety and tolerability, defined by the occurrence of grade 3 and grade 4 possibly related adverse events (PRAEs) within 6 months of treatment initiation. Secondary endpoints included median PFS, 6-month and 12-month PFS, median OS and 12-month OS, as well as health-related quality of life.

Description and grading of AEs were performed according to the Common Terminology Criteria for Adverse Events (CTCAE, version 5.0).

Patients underwent tumor response assessments using CT scans at specific time points. The first assessment, which included a whole-body CT scan, was conducted within 4 weeks after completing the third and final cycle of the induction phase; the second was conducted within 4 weeks after the final RT fraction, and further assessments were scheduled every 12 weeks during the maintenance phase.

Tumor response was evaluated by CT scans using the new response evaluation criteria in solid tumors: revised RECIST guideline (version 1.1).^18^

Health-related quality of life was assessed using the standardized, validated EORTC QLQ-C30 (European Organization for Research and Treatment of Cancer Quality of Life Questionnaire 30-item Quality of Life Questionnaire-Core) and EORTC QLQ-Lung Cancer 13 (LC13) self-administered questionnaires.^19^^,^^20^ These questionnaires were administered at 4 specific time points: at baseline, before RT, at the start of maintenance immunotherapy, and at the end of treatment.

### Statistical design

Statistical analyses were conducted using Stata software (release 19.5, StataCorp, College Station, TX, USA). A 1-sided *P* value < .05 was deemed statistically significant for the primary endpoint analysis. Continuous variables were summarized using the mean and SD, or the median and interquartile range (25th-75th percentiles), while categorical variables were presented as counts and percentages. Relevant 2-sided 95% CIs were calculated and used for sample size estimations in future studies.

Safety analyses involved calculating the proportion of patients experiencing grade 3 or 4 PRAEs within 6 months of enrollment, along with their binomial exact 95% CI. These calculations were based on the safety set, including all patients who received at least 1 dose of the study medication. A 1-sided proportion test compared the observed PRAE rate to an acceptable 35%.

Efficacy assessments utilized Kaplan-Meier estimates of cumulative survival, with 95% CI, which were plotted. Median survival times and 95% CI were also determined. Observation periods began at the date of consent and ended either at the occurrence of the event or the last follow-up, whichever came first. A generalized linear regression model with repeated measures was employed to analyze quality of life scores, with calculation of clustered Huber-White robust standard errors to account for intra-patient correlations. Predicted scores and 95% CIs were graphed at each time point.

Data collection utilized an Electronic Data Capture (EDC) system with eCRFs, specifically based on the RedCap platform (RedCap 14.0.15 - © 2024 Vanderbilt University) at the Fondazione IRCCS Policlinico San Matteo Hospital, managed by the Biostatistics and Clinical Trial Center.

The planned sample size of 45 patients was based on an expected PRAE rate of 20%, which yields an upper CI limit of 34.6% (9.6%-34.6%); therefore a PRAE rate not exceeding 35% within 6 months was considered acceptable (primary endpoint met).

For secondary endpoints, a cohort of 45 patients would enable detection of a 20% difference in 12-month PFS in favor of the experimental treatment, assuming a 50% PFS with standard therapy (PACIFIC data), with 87% power and a 1-sided alpha of 0.05.

The failure to reach the required sample size weakens the statistical reliability of the data, preventing any conclusions based on the estimates made. Nonetheless, the data on the 25 enrolled patients provide valuable insights into the potential feasibility and efficacy of the entire sequence, and we therefore report them in full as they are useful and informative for the design of future studies.

## Results

Between February 2022 and August 2024, 28 patients were screened, and 25 were enrolled across 3 Italian sites. Enrollment was halted earlier than anticipated due to a low accrual rate; the planned sample size of 45 patients was therefore reduced to 25 patients.

Patients’ characteristics are summarized in Table 1. Median age was 70.4 (range: 51 to 84) years; most were of male gender (72%), current (36%) or former (64%) smokers, PD-L1 positive (60%), and PS 0 (32%) or 1 (60%). Squamous and non-squamous histologies were equally represented; 32% of enrolled patients had stage IIIA, 40% had stage IIIB, and 16% had stage IIIC NSCLC.

As required by the protocol, all patients began RT within 6 weeks of their last chemo-immunotherapy dose and started maintenance immunotherapy within 6 weeks of their previous RT dose.

As of data cutoff (August 2024), the median follow-up duration was 15.4 months. All patients received at least 1 infusion of durvalumab. Of these, 18 (72%) did not experience disease progression or AEs ( after induction and receiving RT). Additionally, 16 patients (64%) received at least 1 dose of consolidation durvalumab after RT. Overall, 8 patients (32%) completed the full treatment protocol (12 months of consolidation durvalumab), while 2 (8%) continued treatment, and 15 (60%) discontinued treatment; disease progression (11 patients; 73.3%) and AEs (2 patients; 13.3%) were the most common reasons for discontinuation.

### Safety

Seven patients out of 25 experienced at least 1 grade 3-4 PRAE, representing 28% (95% CI = 10 to 46, *P* = .77). Among all 15 grade 3-4 AEs, 9 were reported as grade 3-4 PRAEs, accounting for 60.0% of grade 3-4 AEs and 6.4% of total AEs. One was linked to durvalumab (cutaneous rash), 8 to chemotherapy, and none to radiation therapy. We did not record any pneumonitis.

Overall, 141 AEs were documented, categorized as follows: 85 (60.3%) grade 1, 41 (29.1%) grade 2, 14 (9.9%) grade 3, and only 1 (0.7%) grade 4; no grade 5 events were noted. Just 1 patient (4%) experienced a grade 4 AE (sepsis). Eleven AEs (7.8%) were classified as serious adverse events (SAEs), specifically 4 grade 2 SAE, 6 grade 3 SAE, and 1 grade 4 SAE. Of these, 81.8% required hospitalization, 64.1% recovered, and 35.9% did not. Overall, 4.5% of patients experienced adverse events of special interest (AESIs). A detailed description of AEs and PRAEs is summarized in Tables 2 and 3.

**Table 2.** List of observed and any grade adverse events.

Adverse event	Any grade, No. (%)	G1, No. (%)	G2, No. (%)	G3, No. (%)	G4, No. (%)
All events	141 (100)	85 (60.3)	41 (29.1)	14 (9.9)	1 (0.7)
Asthenia	15 (10.6)	10 (11.8)	4 (9.8)	1 (7.1)	0 (0)
Fever	11 (7.8)	10 (11.8)	1 (2.4)	0 (0)	0 (0)
Hypomagnesia	8 (5.7)	6 (7.2)	1 (2.4)	1 (7.1)	0 (0)
Arthralgias	6 (4.3)	5 (5.9)	1 (2.4)	0 (0)	0 (0)
Rash	5 (3.5)	3 (3.5)	1 (2,4)	1 (7.1)	0 (0)
Mucositis	5 (3.5)	4 (4.8)	1 (2.4)	0 (0)	0 (0)
Neutropenia	5 (3.5)	1 (1.2)	2 (4.8)	3 (21.3)	0 (0)
Cough	5 (3.5)	3 (3.5)	2 (4.8)	0 (0)	0 (0)
Dyspnea	5 (3.5)	4 (4,8)	1 (2.4)	0 (0)	0 (0)
Lack of appetite	4 (2.8)	3 (3.5)	1 (2.4)	0 (0)	0 (0)
Sloping edema	4 (2.8)	3 (3.5)	1 (2.4)	0 (0)	0 (0)
Anemia	4 (2.8)	1 (1.2)	1 (2.4)	2 (14.2)	0 (0)
Skin disorders/dry skin	4 (2.8)	4 (4.8)	0 (0)	0 (0)	0 (0)
Diarrhea	3 (2.1)	1 (1.2)	2 (4.8)	0 (0)	0 (0)
Sars-2-COVID infection	3 (2.1)	3 (3.5)	0 (0)	0 (0)	0 (0)
Thrombocythemia	3 (2.1)	0 (0)	3 (7.2)	0 (0)	0 (0)
Chest pain	3 (2.1)	2 (2.4)	1 (2.4)	0 (0)	0 (0)
Hypotension	2 (1.1)	0 (0)	2 (4.8)	0 (0)	0 (0)
Pain	2 (1.1)	2 (2.4)	0 (0)	0 (0)	0 (0)
Pneumonia	2 (1.1)	0 (0)	2 (4.8)	0 (0)	0 (0)
Reactions to contrast infusion	2 (1.1)	1 (1.2)	1 (2.4)	0 (0)	0 (0)
Itching	2 (1.1)	2 (2.4)	0 (0)	0 (0)	0 (0)
Respiratory failure	2 (1.1)	0 (0)	1 (2.4)	1 (7.1)	0 (0)
Alopecia	1 (0.7)	0 (0)	1 (2.4)	0 (0)	0 (0)
Anorexia	1 (0.7)	1 (1.2)	0 (0)	0 (0)	0 (0)
Conjunctivitis	1 (0.7)	1 (1.2)	0 (0)	0 (0)	0 (0)
COPD exacerbation	1 (0.7)	0 (0)	1 (2.4)	0 (0)	0 (0)
Esophagitis	1 (0.7)	0 (0)	0 (0)	0 (0)	0 (0)
Fracture of the right hemipelvis	1 (0.7)	1 (1.2)	0 (0)	0 (0)	0 (0)
Gingivitis	1 (0.7)	1 (1.2)	0 (0)	0 (0)	0 (0)
Hypokalemia	1 (0.7)	1 (1.2)	0 (0)	0 (0)	0 (0)
Inflammatory parenchymal thickening	1 (0.7)	0 (0)	1 (2.4)	0 (0)	0 (0)
Leukocytosis	1 (0.7)	1 (1.2)	0 (0)	0 (0)	0 (0)
Sciatica pain	1 (0.7)	0 (0)	1 (2.4)	0 (0)	0 (0)
Pollakiuria	1 (0.7)	1 (1.2)	0 (0)	0 (0)	0 (0)
Acute coronary syndrome	1 (0.7)	0 (0)	0 (0)	1 (7.1)	0 (0)
Acute kidney injury	1 (0.7)	1 (1.2)	0 (0)	0 (0)	0 (0)
Arthritis	1 (0.7)	0 (0)	1 (2.4)	0 (0)	0 (0)
Hypercreatininemia	1 (0.7)	1 (1.2)	0 (0)	0 (0)	0 (0)
Hypertension	1 (0.7)	1 (1.2)	0 (0)	0 (0)	0 (0)
Dysphagia	1 (0.7)	0 (0)	0 (0)	1 (7.1)	0 (0)
Dysphonia	1 (0.7)	1 (1.2)	0 (0)	0 (0)	0 (0)
Epigastralgia	1 (0.7)	0 (0)	1 (2.4)	0 (0)	0 (0)
Fatigue	1 (0.7)	1 (1.2)	0 (0)	0 (0)	0 (0)
Heartburn	1 (0.7)	1 (1.2)	0 (0)	0 (0)	0 (0)
Hyperkalemia	1 (0.7)	0 (0)	1 (2.4)	0 (0)	0 (0)
Hypocalcemia	1 (0.7)	0 (0)	1 (2.4)	0 (0)	0 (0)
Infection of the jejunostomy	1 (0.7)	0 (0)	1 (2.4)	0 (0)	0 (0)
Hypothyroidism	1 (0.7)	1 (1.2)	0 (0)	0 (0)	0 (0)
Ischemic stroke	1 (0.7)	0 (0)	0 (0)	1 (7.1)	0 (0)
Odynophagia	1 (0.7)	1 (1.2)	0 (0)	0 (0)	0 (0)
Metasteroid diabetes	1 (0.7)	0 (0)	1 (2.4)	0 (0)	0 (0)
Pancytopenia	1 (0.7)	0 (0)	0 (0)	1 (7.1)	0 (0)
Paresthesia	1 (0.7)	1 (1.2)	0 (0)	0 (0)	0 (0)
Pneumonia ab ingestis	1 (0.7)	0 (0)	0 (0)	1 (7.1)	0 (0)
Sepsis	1 (0.7)	0 (0)	0 (0)	0 (0)	1 (100.0)
Soft tissue infection	1 (0.7)	0 (0)	1 (2.4)	0 (0)	0 (0)
Constipation	1 (0.7)	1 (1.2)	0 (0)	0 (0)	0 (0)
Worsening of mental confusion	1 (0.7)	0 (0)	1 (2.4)	0 (0)	0 (0)

**Table 3.** List of possibly related adverse events (PRAEs).

Possibly related adverse event (PRAE)	Any grade, No. (%)	G1, No. (%)	G2, No. (%)	G3, No. (%)	G4, No. (%)
All events	53 (100)	30 (56.6)	14 (26.4)	8 (15.1)	1 (1.9)
Asthenia	9 (17.0)	6 (20.7)	2 (14.3)	1 (12.5)	0 (0)
Neutropenia	5 (9.5)	0 (0)	2 (14.3)	2 (25.0)	0 (0)
Hypomagnesemia	5 (9.5)	4 (13.8)	0 (0)	1 (12.5)	0 (0)
Anemia	4 (7.5)	1 (3.4)	1 (7.1)	2 (25.0)	0 (0)
Rash	3 (5.7)	3 (10.3)	0 (0)	1 (12.5)	0 (0)
Mucositis	3 (5.7)	2 (6.9)	1 (7.1)	0 (0)	0 (0)
Arthralgia	3 (5.7)	3 (10.3)	0 (0)	0 (0)	0 (0)
Lack of appetite	3 (5.7)	2 (69)	1 (7.1)	0 (0)	0 (0)
Skin disorders/dry skin	2 (3.8)	2 (6.9)	0 (0)	0 (0)	0 (0)
Itching	2 (3.8)	2 (6.9)	0 (0)	0 (0)	0 (0)
Alopecia	1 (1.9)	0 (0)	1 (7.1)	0 (0)	0 (0)
Anorexia	1 (1.9)	1 (3.4)	0 (0)	0 (0)	0 (0)
Diarrhea	1 (1.9)	0 (0)	1 (7.1)	0 (0)	0 (0)
Esophagitis	1 (1.9)	1 (3.4)	0 (0)	0 (0)	0 (0)
Pneumonia	1 (1.9)	0 (0)	1 (7.1)	0 (0)	0 (0)
Arthritis	1 (1.9)	0 (0)	1 (7.1)	0 (0)	0 (0)
Epigastralgia	1 (1.9)	0 (0)	1 (7.1)	0 (0)	0 (0)
Fatigue	1 (1.9)	1 (3.4)	0 (0)	0 (0)	0 (0)
Hypothyroidism	1 (1.9)	1 (3.4)	0 (0)	0 (0)	0 (0)
Pancytopenia	1 (1.9)	0 (0)	0 (0)	1 (12.5)	0 (0)
Thrombocythemia	1 (1.9)	0 (0)	1 (7.1)	0 (0)	0 (0)
Respiratory failure	1 (1.9)	0 (0)	1 (7.1)	0 (0)	0 (0)
Sepsis	1 (1.9)	0 (0)	0 (0)	0 (0)	1 (100.0)
Constipation	1 (1.9)	1 (3.4)	0 (0)	0 (0)	0 (0)

Lymphopenia data were collected but not considered as clinically significant by investigators because of the high incidence of lymphopenia during and after thoracic RT, without significant influence of this condition on the treatment course. However, data of lymphopenia were collected at 4 specific timepoints: at the beginning of RT, corresponding to cycle 4 of immunotherapy, at the end of RT (cycle 5), 4 weeks after the end of RT (cycle 6) and 8 weeks after the end of RT (cycle 7). At the beginning of RT, 12/18 patients (66.6%) had no lymphopenia, 6/18 patients (33.3%) had grade 1 lymphopenia; at the end of RT, 2/18 patients (11.1%) had grade 1 lymphopenia, 9/18 patients (50%) had grade 2 lymphopenia, and 7/18 patients (38.9%) had grade 3-4 lymphopenia; 4 weeks after the end of RT, 1/16 patients (6.3%) had no lymphopenia, 6/16 patients (37.5%) had grade 1 lymphopenia, 4/16 patients (25%) had grade 2 lymphopenia, and 5/16 patients (31.2%) had grade 3 to 4 lymphopenia; 8 weeks after the end of RT, 10/16 patients (62.5%) had grade 1 lymphopenia, 5/16 patients (31.2%) had grade 2 lymphopenia, and 1/16 patients (6.3%) had grade 3-4 lymphopenia.

### Discontinuation

**Figure 2.**
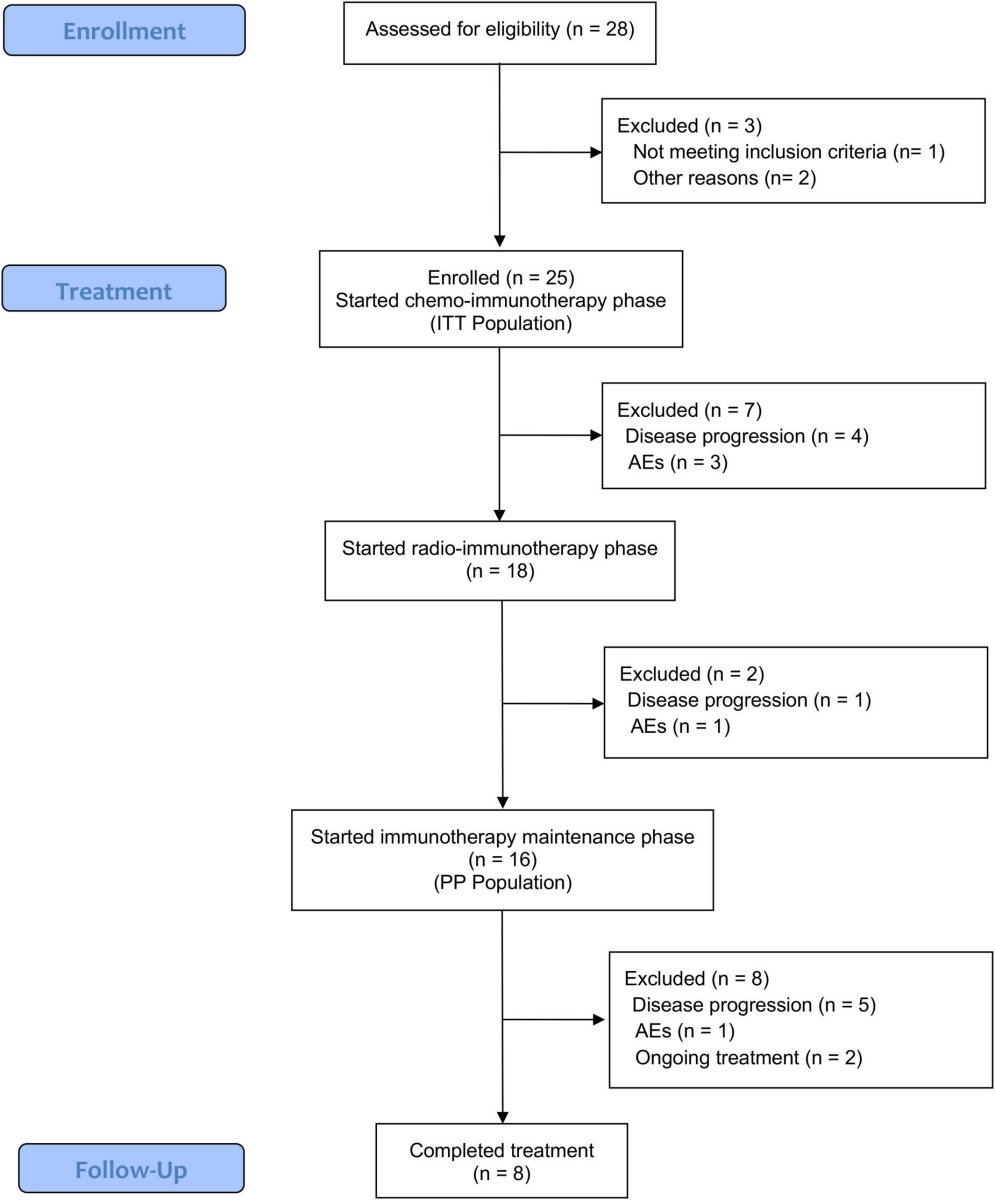
CONSORT diagram showing the flow of participants through each stage of the DEDALUS trial.

Three patients (12%) discontinued chemo-immunotherapy due to severe AEs. For the same reason, 1 patient (4%) temporarily interrupted durvalumab during the consolidation phase, and 1 patient (8%) discontinued it (Figure 2).

### Efficacy

**Figure 3.**
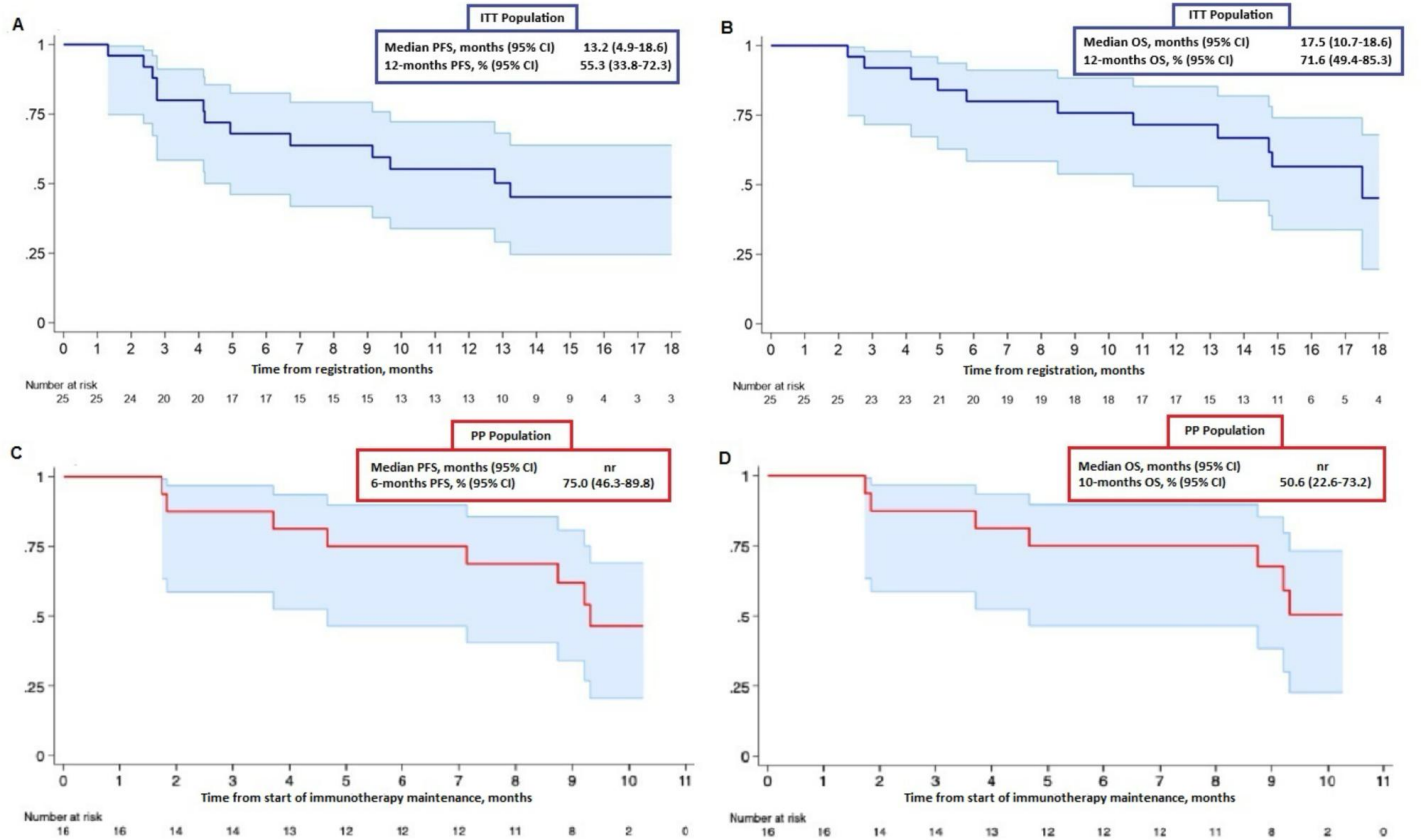
Kaplan-Meier distributions for PFS **(A)** and OS **(B)** in the ITT population, and PFS **(C)** and OS **(D)** in the per-protocol (PP) population. ITT population: PFS is defined as the time from the first dose of chemo-durvalumab (cycle 1) to the date of objective disease progression or death (by any cause in the absence of progression), regardless of whether the patient discontinues treatment or receives another anticancer therapy before progression. OS is defined as the time from the first dose of chemo-durvalumab (cycle 1) to death from any cause. PP population: PFS is defined as the time from the first dose of durvalumab maintenance (cycle 6) to the date of objective disease progression or death (by any cause in the absence of progression), regardless of whether the patient discontinues treatment or receives another anticancer therapy before progression. OS is defined as the time from the first dose of durvalumab maintenance (cycle 6) to death from any cause. Abbreviations: nr = not reached; OS = overall survival; PFS = progression-free survival.

Sixteen patients (64%) experienced disease progression. The median PFS in the intention-to-treat (ITT) population (all 25 patients) was 13.2 months (95% CI = 4.9 to 18.6); 12-month cumulative PFS was 55.3% (95% CI = 33.8 to 72.3) (Figure 3A).

Overall, 15 patients (60%) died, and the median follow-up duration among patients censored for OS was 15.4 months. Median OS in the ITT population was 17.5 months (95% CI = 10.7 to 18.6). The 12-month cumulative OS was 71.6% (95% CI = 49.4 to 85.3) (Figure 3B).

Figure 3C and D present an exploratory landmark analysis of 16 patients who completed RT (per-protocol, PP), with observation beginning in the first cycle of immunotherapy maintenance (PFS in Figure 3C and OS in Figure 3D).

Additionally, the Supplementary Methods (Figure S1A and B) display PFS and OS outcomes for this subset, revealing that when observation begins at C1, the median PFS in the PP population is 18.6 months (95% CI = 12.8 to not reached). The 12-month cumulative PFS is 81.3% (95% CI = 51.5 to 93.5) (Figure S1A), while median OS has not yet been reached. The 18-month cumulative OS is 68.1% (95% CI = 25.1 to 89.9) (Figure S1B).

**Figure 4.**
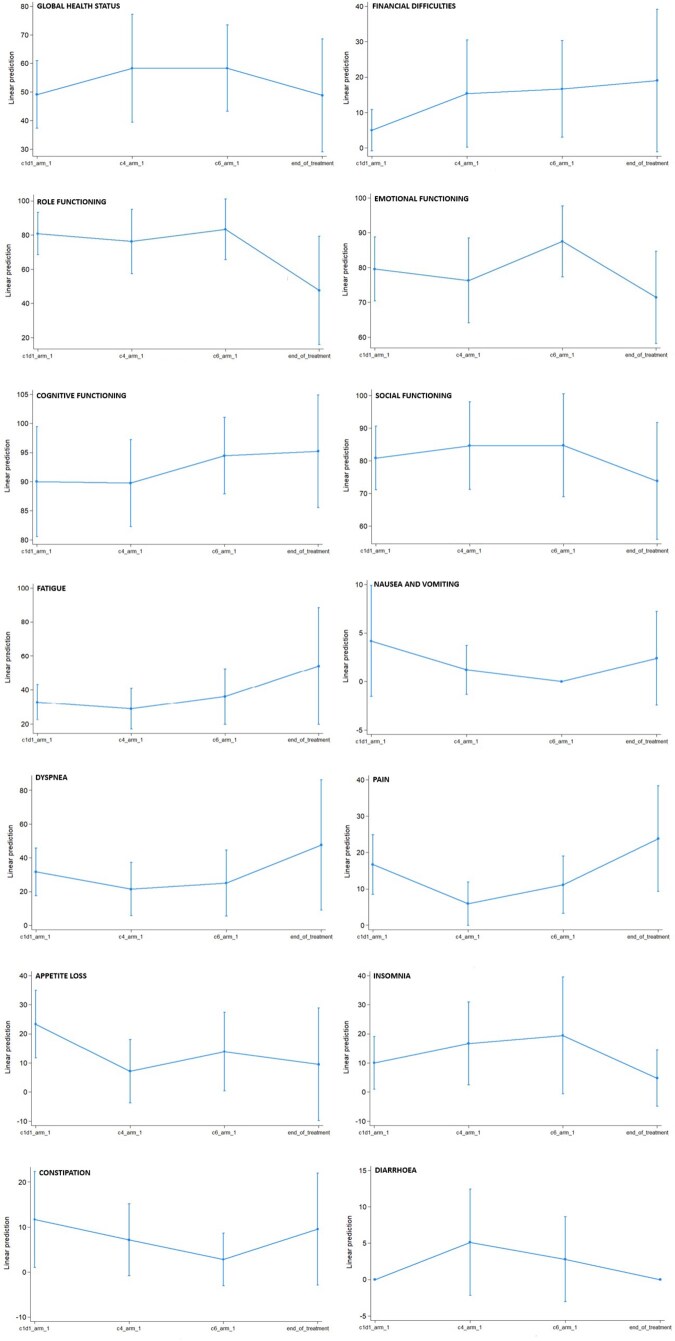
Changes from baseline in global health status (GHS) and functional symptoms, based on the EORTC QLQ C30 questionnaire. It was administered at 4 specific timepoints: at baseline (start of chemo-immunotherapy), at the beginning of radio-immunotherapy, at the start of maintenance immunotherapy, and at the end of treatment.

**Figure 5.**
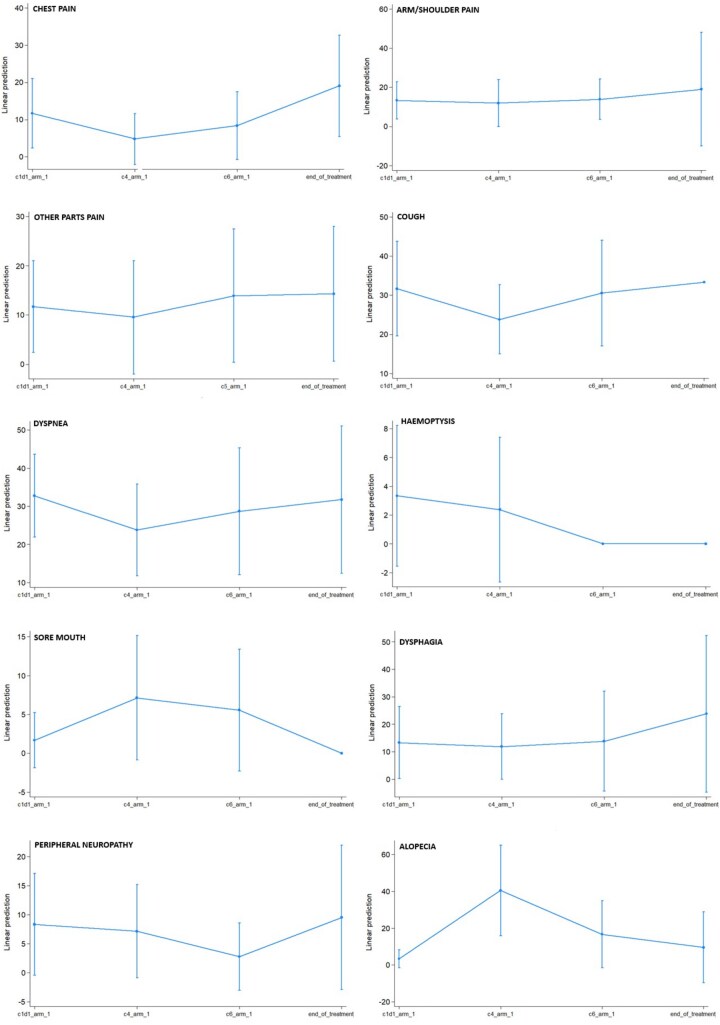
Evolution from baseline specific lung cancer functional symptoms, based on EORTC LC13 questionnaire. It was administered at 4 specific timepoints: at baseline (beginning of chemo-immunotherapy), at the beginning of radio-immunotherapy, at the beginning of maintenance immunotherapy, and at the end of treatment.

Twenty patients (80%) completed the EORTC QLQ C30 and LC13 questionnaires at baseline, 14 (56%) by the start of RT, 12 (48%) by the start of maintenance immunotherapy, and 7 (28%) at the end of treatment. The evolution of global health status (GHS) and functional symptoms during follow-up is shown in Figures 4 and 5.

Statistically significant changes were observed during the study treatment only for improved emotional functioning (*P* = .014), a borderline non-significant worsening of appetite loss (*P* = .088), as assessed by the EORTC QLQ C30, and an increase in alopecia (*P* = .012), as evaluated by the LC13. No statistically significant changes were seen during the treatment concerning other quality of life parameters; during follow-up, we observed overall stability in GHS score, fatigue, dyspnea, pain control, and dysphagia, along with a consistent trend toward improvement in role functioning, social functioning, hemoptysis, and sore mouth.

## Discussion

The phase 2 nonrandomized DEDALUS trial assessed the safety and effectiveness of a new treatment approach for patients with unresectable stage III NSCLC who are not suitable for cCRT followed by immunotherapy (IO). The goal of this new regimen was to improve the current standard of care by using induction chemo-immunotherapy to enhance local and systemic disease control, while reducing RT doses to minimize side effects and maintain the same level of disease control and survival. Immunotherapy administered early in the treatment course, before the onset of radiation-induced lymphopenia, may enhance immune priming and the anti-tumor response on both sequential/concurrent CRT. Furthermore, reducing radiation dose could help preserve immune function, mitigate radiation-induced immunosuppression, and lower the risk of late toxicities, including esophagitis and pneumonitis. New onset of grade 3-4 lymphopenia was observed in 38.9% of patients after the end of RT; this percentage reduced after 4 weeks and 8 weeks (31.2% and 6.3%, respectively), suggesting a rapid restoration of lymphocyte-mediated immunological competence after the end of RT and the beginning of maintenance immunotherapy. The safety profile observed in our study aligned with expectations for combination chemo-immunotherapy, with most AEs being manageable. Grade 3-4 PRAEs occurred in 28.0% of patients, mainly caused by CT. Only 1 grade 3-4 event was linked to durvalumab, and none were linked to RT, indicating that the reduced-dose RT was well tolerated in this setting. Importantly, no pneumonitis and no grade 5 events were reported.

The median PFS for the ITT cohort (*n* = 25) was 13.2 months, with a 12-month PFS rate of 55.3%. In both the landmark analysis starting from the end of RT (Figure 3B and C) and at the beginning of observation at C1 (Figure 1A and B, Supplementary Material), patients who completed RT (defined as per-protocol population (*n* = 16)) demonstrated longer PFS and OS. However, these comparisons are not conclusive because the PP analysis is exploratory and includes only patients who completed RT, potentially introducing survival bias.

Exploratory benchmark comparisons can be made using safety and efficacy data from the PACIFIC-6 trial,^13^^,^^14^ which employed nearly identical inclusion and exclusion criteria. Grade 3-4 AEs were observed in 18.8% of 117 PACIFIC-6 participants, while PRAEs within 6 months were reported in 4.3%, including 2 cases of pneumonitis; grade 5 events occurred in 1.7% of the cohort. In our study, the overall PRAE rate was 28.0% (7 patients). Still, only 1 was immune-related, and none were RT-related, possibly reflecting differences in treatment sequencing, RT dosing, or baseline patient characteristics. The median PFS in PACIFIC-6 was 10.9 months (95% CI = 7.3 to 15.6), with 12-month PFS and OS rates of 49.6% and 84.1%, respectively. In our ITT population, the median PFS was 13.2 months, with a similar 12-month PFS rate of 55.3%. Notably, accounting for statistical limitations, median PFS in the DEDALUS PP population was longer, suggesting that early induction and RT de-escalation could improve outcomes in selected patients.

Front-loading immunotherapy during induction CT could be crucial for the regimen’s success. However, combining anti-PD-L1 upfront with concurrent chemo-radiotherapy has demonstrated disappointing results in the PACIFIC-2 trial,^21^ CheckMate 73L trial,^22^ and also in limited-stage small cell lung cancer.^23^ As a result, there is increasing interest in administering chemo-immunotherapy before radiation to improve responses without raising toxicity, reduce treatment volume, and potentially extend PFS.^24^ Several phase 2, single-arm trials involving patients suitable for the PACIFIC regimen have already reported promising outcomes: the PACIFIC Brazil trial,^25^ the APOLO trial,^26^ and the AFT-16 trial.^27^ The last used immunotherapy alone as induction in patients overexpressing PD-L1.

To our knowledge, DEDALUS is the first trial to test this approach in a more vulnerable population, specifically candidates for sequential CRT plus immunotherapy, incorporating RT de-escalation. It has shown promising results and supports the evolving idea that carefully reducing RT in selected patients may maintain tumor control. The early trial closure decreased the sample size from 45 to 25, thereby lowering the statistical power for safety and efficacy endpoints. Moreover, the lack of a control arm limits direct comparison with standard treatments. It is difficult to draw significant conclusions from this study because of the sample size and the low completion rate (around 30%-35%); this is an early phase 2 study that should act as a guide for the design of future comparative studies and, therefore, caution should be exercised when interpreting efficacy results. The main reasons for not completing the regimen were disease progression for 10 patients and treatment-related toxicity (most due to CT) for 5 patients, emphasizing the importance of patient selection for success in sequential approaches for frail patients. Future studies should incorporate biomarkers and multidimensional baseline assessments to more accurately predict toxicity risk, which may be crucial for developing effective strategies, especially in frailer patients.

The stability of most HRQoL parameters indicates this approach is acceptable from the patient’s perspective, especially considering the extended treatment period in a potentially curative setting. In particular, the lack of significant worsening of QoL, which can be seen during concurrent chemoRT, could be considered the major point of the patient reported outcomes (PRO) collected in this study.

In summary, our study shows that induction chemo-immunotherapy followed by reduced-dose hypo-fractionated RT and durvalumab maintenance is a feasible and potentially effective strategy for unresectable stage III NSCLC unsuitable for cCRT followed by immunotherapy. Further validation could transform this into a less toxic, more personalized treatment option that maintains curative intent while enhancing quality of life for selected patients.

## Supplementary Material

pkag050_Supplementary_Data

## Data Availability

The data underlying this article are available in the article and in its online Supplementary Material.

## References

[1] Siegel RL , Miller KD, Wagle NS, et al Cancer statistics, 2023. CA Cancer J Clin. 2023;73:17-48. 36633525 10.3322/caac.21763

[2] Molina JR , Yang P, Cassivi SD, et al Non-small cell lung cancer: epidemiology, risk factors, treatment, and survivorship. Mayo Clinic Proc. 2008;83:584-594. 10.4065/83.5.584 PMC2718421 18452692

[3] Duan L , Cui H, Zhang W, et al Symptoms and experiences of frailty in lung cancer patients with chemotherapy: a mixed-method approach. Front Oncol. 2022;12:1019006. 36276107 10.3389/fonc.2022.1019006 PMC9582838

[4] Daly ME , Singh N, Ismaila N, et al Management of stage III non-small cell lung cancer ASCO guidelines. J Clin Oncol. 2022;40:1356-1384. 34936470 10.1200/JCO.21.02528

[5] Remon J , Soria JC, Peters S; ESMO Guidelines Committee. Early and locally advanced non-small cell lung cancer: an update of the ESMO Clinical Practice Guidelines focusing on diagnosis, staging, systemic and local therapy. Ann Oncol 2021;32:1637-1642. 34481037 10.1016/j.annonc.2021.08.1994

[6] Antonia SJ , Villegas A, Daniel D, et al; PACIFIC Investigators. Durvalumab after chemoradiotherapy in stage III non-small cell lung cancer. N Engl J Med. 2017;377:1919-1929. 28885881 10.1056/NEJMoa1709937

[7] Antonia SJ , Villegas A, Daniel D, et al; PACIFIC Investigators. Overall survival with durvalumab after chemoradiotherapy in stage III NSCLC. N Engl J Med. 2018;379:2342-2350. 30280658 10.1056/NEJMoa1809697

[8] Spigel DR , Faivre-Finn C, Gray JE, et al Five-year survival outcomes from the PACIFIC trial: durvalumab after chemoradiotherapy in stage III non-small cell lung cancer. J Clin Oncol. 2022;40:1301-1311. 35108059 10.1200/JCO.21.01308 PMC9015199

[9] Kordbacheh T , Honeychurch J, Blackhall F, et al Radiotherapy and PD-1/PD-L1 combinations in lung cancer: building better translational research platforms. Ann Oncol. 2018;29:301-310. 29309540 10.1093/annonc/mdx790

[10] Demaria S , Golden EB, Formenti SC. Role of local radiation therapy in cancer immunotherapy. JAMA Oncol. 2015;1:1325-1332. 26270858 10.1001/jamaoncol.2015.2756

[11] Girard N , Bar J, Garrido P, et al Treatment characteristics and real-world progression-free survival in patients with unresectable stage III NSCLC who received durvalumab after chemoradiotherapy: findings from the PACIFIC-R study. J Thorac Oncol. 2023;18:181-193. 36307040 10.1016/j.jtho.2022.10.003

[12] Filippi AR , Bar J, Chouaid C, et al Real-world outcomes with durvalumab after chemoradiotherapy in patients with unresectable stage III NSCLC: interim analysis of overall survival from PACIFIC-R. ESMO Open 2024;9(6):103464. 10.1016/j.esmoop.2024.103464 PMC11179087 38833971

[13] Garassino MC , Mazieres J, Reck M, et al Durvalumab after sequential chemoradiotherapy in stage III, unresectable NSCLC: the phase 2 PACIFIC-6 trial. J Thorac Oncol. 2022;17:1415-1427. 35961520 10.1016/j.jtho.2022.07.1148

[14] Garassino MC , Faivre-Finn C, Putora PM, et al Safety and efficacy of durvalumab after sequential chemoradiotherapy in unresectable stage III NSCLC: the PACIFIC-6 phase II trial. Ann Oncol. 2022;33:S1400-S1401.

[15] Zhou Q , Chen M, Jiang O, et al Sugemalimab versus placebo after concurrent or sequential chemoradiotherapy in patients with locally advanced, unresectable, stage III non-small-cell lung cancer in China (GEMSTONE-301): interim results of a randomised, double-blind, multicentre, phase 3 trial. Lancet Oncol. 2022;23:209-219. 35038429 10.1016/S1470-2045(21)00630-6

[16] Detterbeck FC , Boffa DJ, Kim AW, et al The eighth edition lung cancer stage classification. Chest. 2017;151:193-203. 27780786 10.1016/j.chest.2016.10.010

[17] World Medical Association. World Medical Association Declaration of Helsinki: ethical principles for medical research involving human subjects. JAMA. 2013;310:2191-2194. 24141714 10.1001/jama.2013.281053

[18] Eisenhauer EA , Therasse P, Bogaerts J, et al New response evaluation criteria in solid tumors: revised RECIST guideline (version 1.1). Eur J Cancer. 2009;45:228-247. 19097774 10.1016/j.ejca.2008.10.026

[19] Aaronson NK , Ahmedzai S, Bergman B, et al The European Organization for Research and Treatment of Cancer QLQ-C30: a quality-of-life instrument for use in international clinical trials in oncology. J Natl Cancer Inst. 1993;85:365-376. 8433390 10.1093/jnci/85.5.365

[20] Bergman B , Aaronson NK, Ahmedzai S, et al The EORTC QLQ-LC13: a modular supplement to the EORTC Core Quality of Life Questionnaire (QLQ-C30) for use in lung cancer clinical trials. EORTC Study Group on Quality of Life. Eur J Cancer. 1994;30A:635-642. 8080679 10.1016/0959-8049(94)90535-5

[21] Bradley JD , Sugawara S, Lee KHH, et al Durvalumab in combination with chemoradiotherapy for patients with unresectable stage III NSCLC: final results from PACIFIC-2. ESMO Open. 2024;9:102986.

[22] De Ruysscher D , Ramalingam S, Urbanic J III, et al CheckMate 73L: a phase 2 study comparing nivolumab plus concurrent chemoradiotherapy followed by nivolumab with or without ipilimumab versus concurrent chemoradiotherapy followed by durvalumab for previously untreated, locally advanced stage non-small cell lung. Clin Lung Cancer 2022;23:e264-e268. 34489161 10.1016/j.cllc.2021.07.005

[23] Higgins K , Hu C, Ross HJ, et al Concurrent chemoradiation +/− atezolizumab (atezo) in limited-stage small cell lung cancer (LS-SCLC): results of NRG Oncology/Alliance LU005. J Clin Oncol. 2026;44:630–640. 41529214 10.1200/JCO-25-01569 PMC12874332

[24] Thawani R , Bestvina C, Vokes E, et al Rationale for investigation of neoadjuvant chemoimmunotherapy before chemoradiation in unresectable stage III non-small cell lung cancer. J Clin Oncol. 2025;43:2039-2043. 40146965 10.1200/JCO-24-02355

[25] Nassib WW Jr , Castro G Jr, Matias D, et al Intensified chemo-immuno-radiotherapy with durvalumab for stage III NSCLC: a Brazilian single arm phase II study – PACIFIC Brazil (LACOG 2218). J Thorac Oncol. 2023;18(3):Supplement 2, S8.

[26] Provencio M , Campas B, Guirado M, et al Phase II trial of induction chemo-immunotherapy plus chemoradiotherapy and maintenance immunotherapy in stage III NSCLC. J Thorac Oncol. 2024;19(10):Supplement S37.

[27] Ross HJ , Kozono D, Wang XF, et al Atezolizumab before and after chemoradiation for unresectable stage III non-small cell lung cancer: a phase II nonrandomized controlled trial. JAMA Oncol. 2024;10:1212-1219. 39052256 10.1001/jamaoncol.2024.1897 PMC11273282

