# Supplementary material for: Sequential chemo-durvalumab, reduced-dose RT, and consolidation durvalumab for unresectable stage III NSCLC unfit for PACIFIC regimen (DEDALUS trial)
Source: JNCI Cancer Spectr. 2026 May 6;10(3):pkag050. doi: 10.1093/jncics/pkag050 (PMC13242927; doi:10.1093/jncics/pkag050)
Supplement: pkag050_Supplementary_Data [file pkag050_supplementary_data.pdf]

Supplementary materials

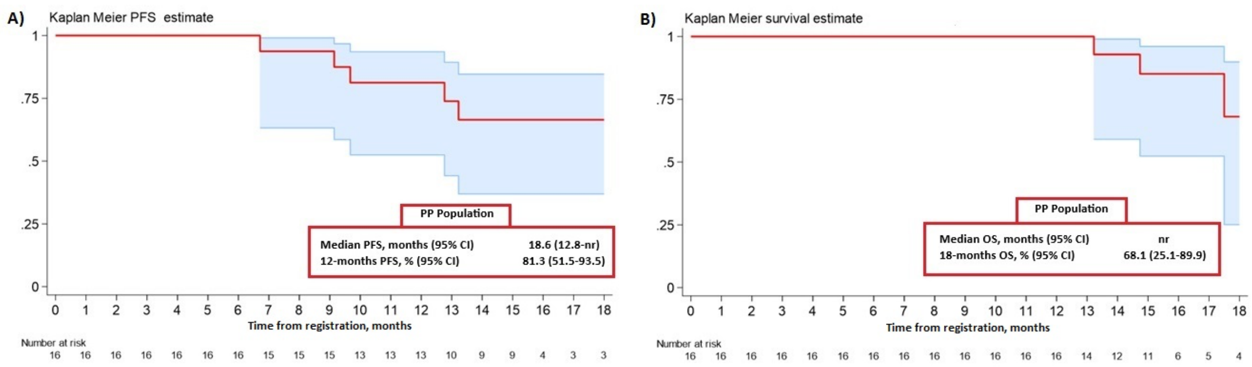

Figure S1. Kaplan-Meier distributions for (A) PFS and (B) OS in the PP population.

PFS is defined as the time from the first dose of chemo-durvalumab to the date of objective disease progression or death (by any cause in the absence of progression), regardless of whether the patient discontinues treatment or receives another anticancer therapy before progression. OS is defined as the time from the first dose of chemo-durvalumab to death from any cause.

CI: confidence interval; NR: not reached; OS: overall survival; PFS: progression-free survival.
